# Monitoring Microbial Populations and Antibiotic Resistance Gene Enrichment Associated with Arctic Waste Stabilization Ponds

**DOI:** 10.1128/AEM.02914-20

**Published:** 2021-03-11

**Authors:** Monica Gromala, Josh D. Neufeld, Brendan J. McConkey

**Affiliations:** a Department of Biology, University of Waterloo, Waterloo, Ontario, Canada; Chinese Academy of Sciences

**Keywords:** Arctic ecology, DNA sequencing, antibiotic resistance, microbial communities, waste stabilization ponds, wastewater

## Abstract

Wastewater management in the Canadian Arctic is challenging due to climate extremes, small population sizes, and lack of conventional infrastructure for wastewater treatment. Although many northern communities use waste stabilization ponds (WSPs) as their primary form of wastewater treatment, few studies have explored WSP microbial communities and assessed effluent impacts on receiving waters from a microbiological perspective. Here, we used 16S rRNA gene and metagenome sequencing to characterize WSP and receiving water microbial communities for two time points bracketing the spring WSP thaw in Baker Lake (Nunavut) and compared these results to other Nunavut WSPs in Cambridge Bay and Kugluktuk. Most amplicon sequence variants (ASVs) recovered from these WSP samples belonged to the phylum *Proteobacteria*, with considerable variation between the three locations and only six ASVs shared among the WSPs at >0.2% relative abundance. Wastewater indicator ASVs for the Baker Lake WSP were identified, and few indicator ASVs were detected in samples originating from other upstream or downstream sites. The metagenomic data revealed a strong enrichment of antibiotic resistance genes for WSP samples relative to downstream and reference samples, especially for genes associated with macrolide resistance. Together, our results provide a baseline characterization for WSP microbial communities, demonstrate how indicator ASVs can be used to monitor attenuation and dilution of effluent microorganisms, and reveal that WSPs can serve as hot spots for antibiotic resistance genes.

**IMPORTANCE** Given that the microbial communities of Arctic waste stabilization ponds (WSPs) are poorly studied to date, our characterization of multiple WSP systems and time points provides important baseline data that will assist with ongoing monitoring of effluent impacts on downstream aquatic ecosystems in the Arctic. This research also identifies indicator amplicon sequence variants (ASVs) of WSPs that will be helpful for future monitoring for WSP effluent attenuation and demonstrates that WSP microbial communities are enriched in antibiotic resistance genes. Given operational and infrastructure changes anticipated for wastewater treatment systems in the Arctic, baseline data such as these are essential for further development of safe and effective wastewater treatment systems.

## Abstract (precis)

Given that the microbial communities of Arctic waste stabilization ponds (WSPs) are poorly studied to date, our characterization of multiple WSP systems and time points provides important baseline data that will assist with ongoing monitoring of effluent impacts on downstream aquatic ecosystems in the Arctic. This research also identifies indicator amplicon sequence variants (ASVs) of WSPs that will be helpful for future monitoring for WSP effluent attenuation and demonstrates that WSP microbial communities are enriched in antibiotic resistance genes.

## INTRODUCTION

As a complex mixture of particulate and dissolved organic matter, nutrients, bacteria, metals, oils, and plastics, municipal wastewater effluent can have multiple impacts on the biological communities of receiving waters (1). When treated inadequately, municipal wastewater effluent can cause eutrophication due to the enrichment of macro- and micronutrients (2) and increased concentrations of nitrogen and phosphorus that promote the growth of harmful algal species (3). Such eutrophication can lead to microbial proliferation, biomass production, oxygen depletion, and toxin release (4). Many studies have documented changes in microbial communities associated with effluent inputs, including phylum-level differences upstream and downstream of wastewater effluent discharge sites (5), increased microbial abundances at sites downstream of discharge sites (6), and downstream persistence of microorganisms from wastewater effluent (7). Additionally, microbial community diversity decreases were seen in downstream sediment (8), and impacted sediment sites were associated with decreased abundances of sulfate reducers, denitrifiers, and methanogens in comparison to sediments from upstream sites (9). Microplastics from wastewater effluent contribute to the formation of microbial biofilms (10), which increases their ability to persist downstream of effluent input.

Because anthropogenic antibiotics are processed through municipal wastewater, conventional wastewater treatment plant systems are recognized as hot spots for the dissemination and evolution of antibiotic resistance genes (ARGs) (11). Antibiotics in wastewater exert selective pressures on microbial communities in these systems (12) and enable further dissemination through horizontal gene transfer of ARG-containing plasmids that are passed from antibiotic-resistant bacteria (ARB) to sensitive recipients (13). Human pathogens may also be present within municipal wastewater, with the potential to acquire ARGs in this way.

Although wastewater treatment facilities have been constructed across temperate regions, including Canada (1), wastewater management in northern communities is limited due to the extreme climate of the Arctic and relatively small population sizes (14, 15). Isolated from the rest of Canada, northern communities also face logistical obstacles to the construction and operation of conventional wastewater treatment plants (WWTPs) (16). Arctic wastewater management solutions aim to prevent the deterioration of water quality and protect communities from exposure to potential pathogens while also minimizing operational and infrastructure costs (14). Currently, many northern Canadian communities use waste stabilization ponds (WSPs), also referred to as sewage lagoons or wastewater lagoons, for wastewater treatment (17, 18). In such small Nunavut communities, wastewater is transported directly from buildings of origin to the designated WSP by truck and, after deposit, freezes in the WSP for approximately 9 months of the year (19). During the other 3 months, which are collectively known as the “treatment season,” higher temperatures and longer periods of sunlight result in a thawing of the waste and subsequent flow into receiving waters (18). Warmer conditions also enable microorganisms to perform aerobic and anaerobic degradation of organic matter found within the wastewater, thus reducing the amount of potentially harmful material entering downstream water bodies (20).

Although studies have profiled microbial communities of Arctic environments (21–27), microorganisms inhabiting Arctic WSPs and downstream bodies of water have not been investigated extensively. A study of a Finnish WSP identified dominant influent bacterial taxa related to the genera *Trichococcus*, *Methylorosula*, *Polaromonas*, and *Arcobacter*, as well as the families *Leptotrichiaceae*, *Comamonadaceae*, *Alcaligenaceae*, and *Holophagaceae* (28), although effluent samples from the wastewater treatment system were not investigated. Another study characterized the microbial communities of effluent water from Pond Inlet WSP in Nunavut, Canada (29), showing that the effluent contained dominant bacteria associated with the genera *Rhodoferax*, *Arcobacter*, and *Pseudomonas*.

Given that microbial communities of Arctic WSPs and their persistence in downstream receiving waters are poorly understood, the objectives of this study were to examine the WSP systems of Baker Lake, Cambridge Bay, and Kugluktuk, all in Nunavut, Canada, with a particular focus on sampling Baker Lake. The goal was to characterize microbial communities represented by the WSP samples, in comparison to those of upstream and downstream sites, and identify indicator taxa that may be monitored for downstream effluent contamination. Given the potential for WSPs to facilitate horizontal transfer of ARGs, we used metagenomics to profile Arctic WSP samples. We anticipate that the work presented here will inform future efforts to increase WSP infrastructure as population sizes continue to grow in these northern communities.

## RESULTS AND DISCUSSION

### Temporal and spatial variability of Baker Lake WSP and downstream sites.

**FIG 1:**
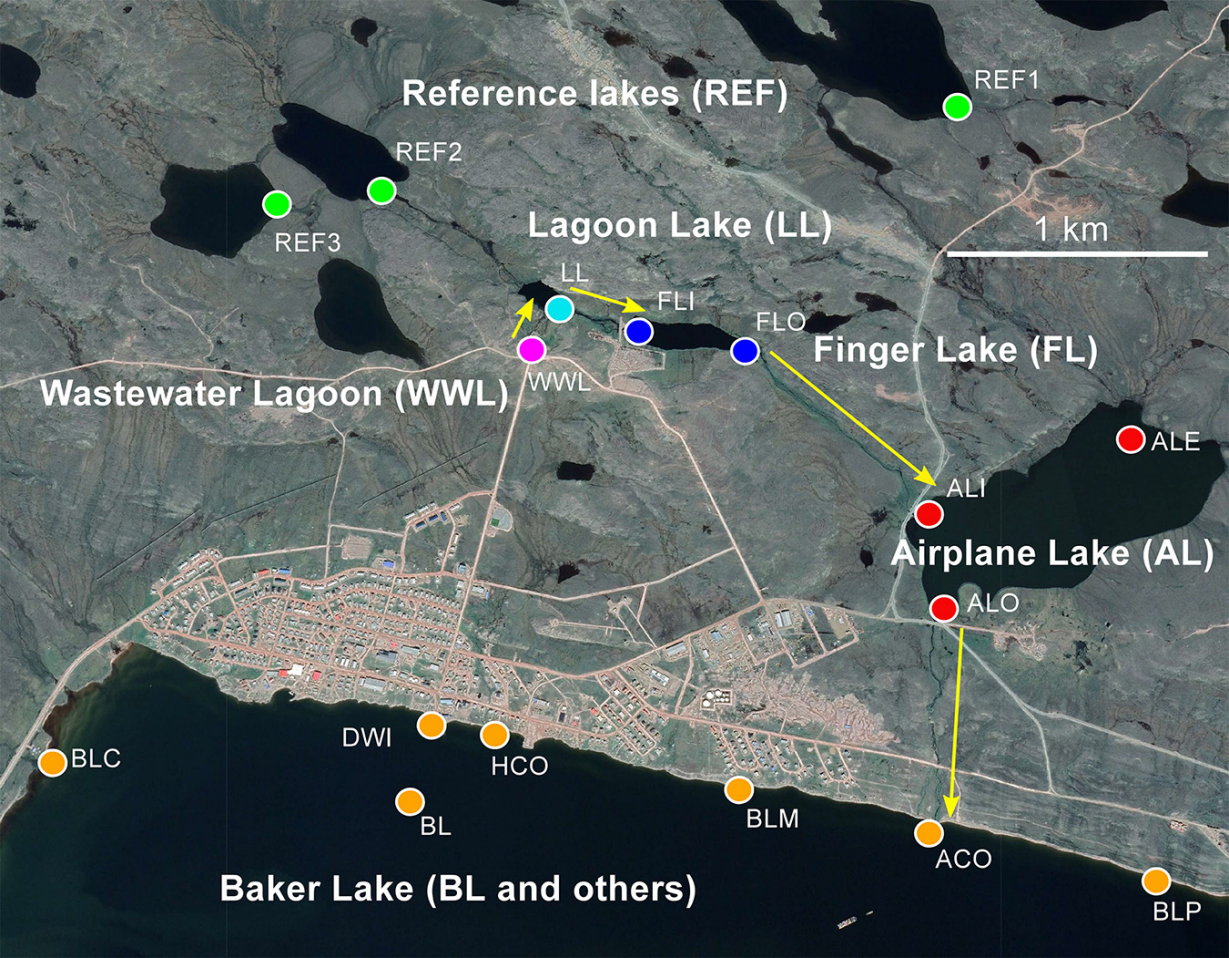
Map of sample sites for Baker Lake, Nunavut, Canada. Samples were grouped by lake as follows and as indicated by the colored circles: Baker Lake waste stabilization pond (WWL; magenta), Lagoon Lake (turquoise), Finger Lake (blue), Airplane Lake (red), Baker Lake (orange), and references (green). WWL, wastewater lagoon; LL, Lagoon Lake; FLI, Finger Lake inlet; FLO, Finger Lake outlet; ALI, Airplane Lake inlet; ALO, Airplane Lake outlet; ALE, Airplane Lake east; ACO, Airplane Creek outlet; BLP, Baker Lake port; BLM, Baker Lake mid; HCO, Hamlet Creek outlet; DWI, drinking water intake; BL, Baker Lake; BLC, Baker Lake Camp; REF1, reference 1; REF2, reference 2; REF3, reference 3. Flow of effluent from WSP is indicated by yellow arrows. The map was adapted from Google Earth.

**FIG 2:**
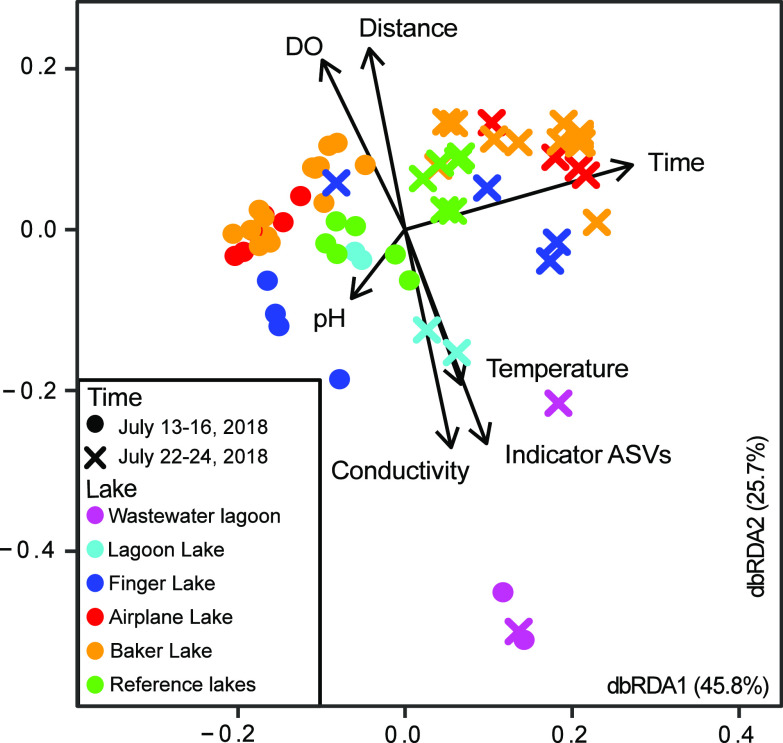
Distance-based redundancy analysis (db-RDA) based on principal-coordinate analysis results and environmental data for sites in Baker Lake, Nunavut, Canada.

Microbial samples were collected in the Baker Lake, vicinity during and after spring thaw on 13 to 16 July 2018 and 22 to 24 July 2018, as well as the Cambridge Bay WSP on 4 July and 25 July 2018 and the Kugluktuk WSP on 16 August 2018. The microbial community composition of water samples from the WSP and downstream receiving waters in Baker Lake (Nunavut; Fig. 1) were influenced by both temporal and spatial factors. Based on an ordination of 16S rRNA gene profiles (Fig. 2), samples collected during the first prethaw time point in Baker Lake (13 to 16 July 2018) separated from those collected during the second time point (22 to 24 July 2018). In addition, samples separated in ordination space based on distance from the WSP; sample sites that were located geographically closer to the WSP appeared closer to WSP samples in ordination space. Sites more distant from the WSP grouped with reference lake samples (Fig. 2). Temperature and other environmental data were also collected (see Table S1 in the supplemental material); however, these measurements did not exhibit a consistent direction of change between the two time points. Temperature, conductivity, dissolved oxygen (DO), and WSP-specific indicator taxa (indicator ASVs; see below) correlated with the separation of samples based on geographic location (Fig. 2).

**FIG 3:**
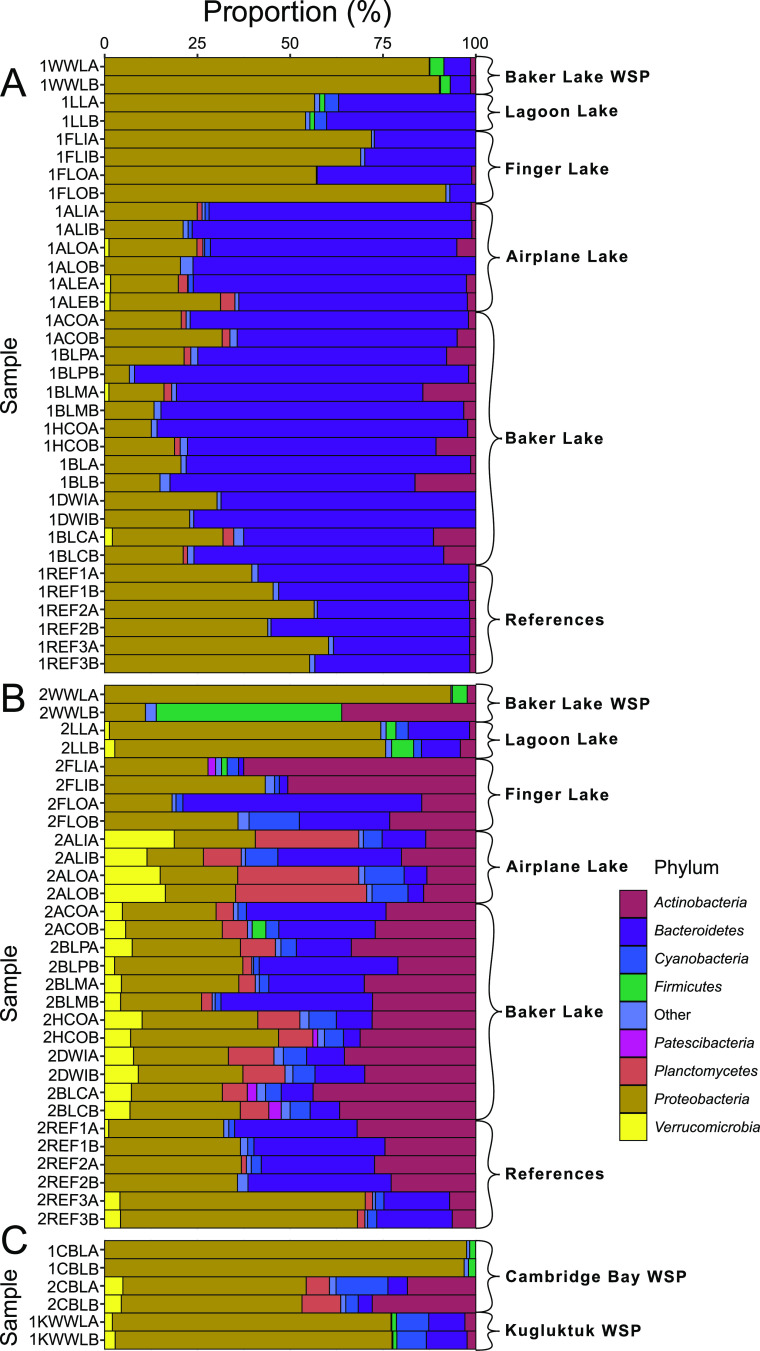
Relative abundance of phyla present at each site in Baker Lake, Cambridge Bay, and Kugluktuk, Nunavut, Canada. (A) Baker Lake, 13 to 16 July 2018; (B) Baker Lake, 22 to 24 July 2018. (C) Cambridge Bay WSP, 4 and 25 July 2018, and Kugluktuk WSP, 18 August 2018. Baker Lake sample names are coded by sites in Fig. 1, with prefixes 1 and 2 indicating sampling time and suffixes A and B indicating sample replicate. Cambridge Bay and Kugluktuk samples are labeled as CBL and KWWL, respectively.

Temporal and spatial variability were apparent within microbial community profiles when assessed at the phylum level (Fig. 3), revealing overall shifts with sampled distance from the WSP. The phylum *Proteobacteria* comprised >80% of the microbial community of the Baker Lake WSP at both time points. In contrast, changes in phylum abundance profiles occurred for the majority of sampled sites over the 10-day period between sampling time points. For example, although the phylum *Bacteroidetes* dominated the first time point samples collected from Airplane Lake and Baker Lake, higher levels of *Actinobacteria*, *Cyanobacteria*, and *Verrucomicrobia* were observed for the second time point samples of these lakes. Finger Lake samples were dominated by *Proteobacteria* for the first time point but then had higher levels of *Actinobacteria* and *Bacteroidetes* later in July 2018. The WSP and Lagoon Lake samples were relatively consistent temporally, with the exception of the 2WWLB (“second time point wastewater lagoon, replicate B”) sample, which contained a high relative abundance of *Firmicutes*.

### Uniqueness of Baker Lake WSP samples.

**FIG 4:**
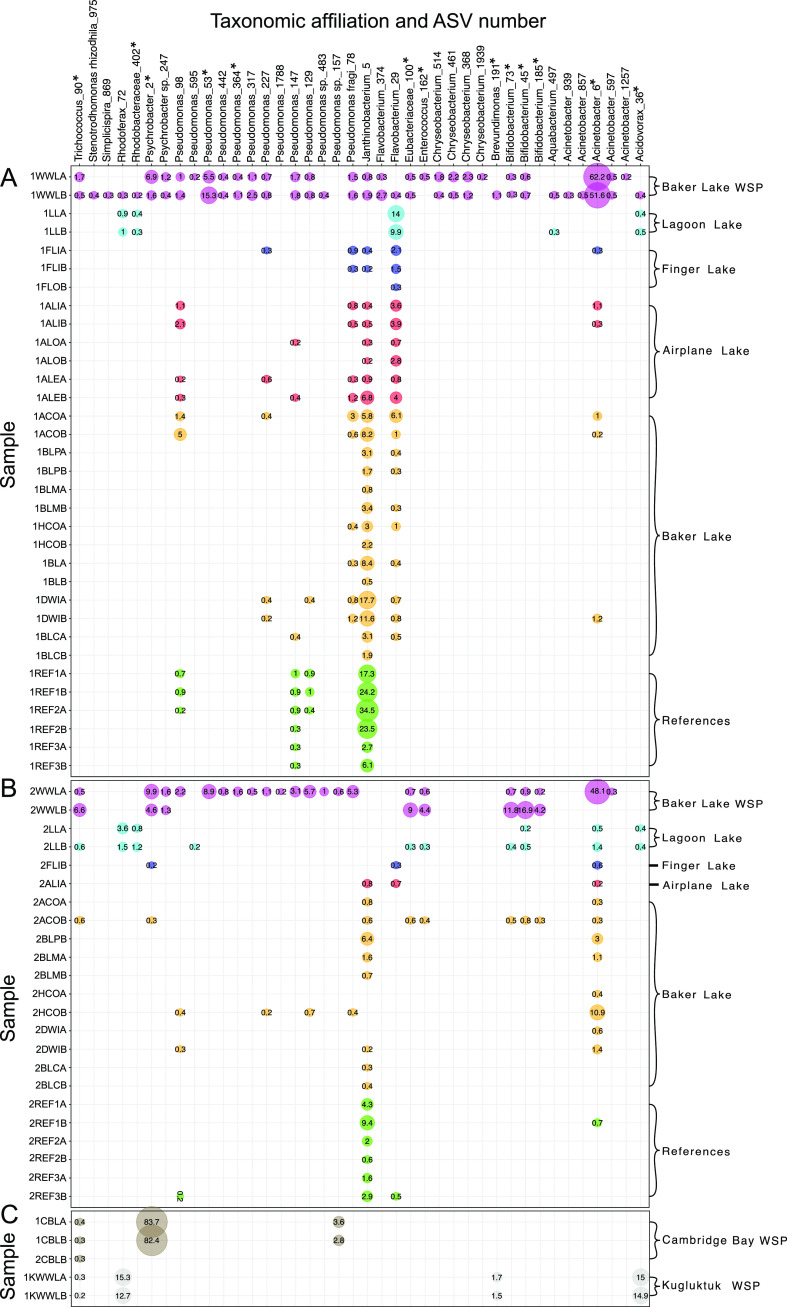
Relative abundance of amplicon sequence variants (ASVs) present at >0.2% abundance at each site in Baker Lake, Cambridge Bay, and Kugluktuk, Nunavut, Canada. (A) Baker Lake, 13 to 16 July 2018. (B) Baker Lake, 22 to 24 July 2018. (C) Cambridge Bay and Kugluktuk. ASVs with <0.2% abundance in 1WWLA, 1WWLB, and 2WWLA were not included. Samples containing >0.2% abundance of any of these ASVs were not included. Baker Lake sample names are coded by sites on Fig. 1, with prefixes 1 and 2 indicating sampling time and suffixes A and B indicating sample replicate. Cambridge Bay and Kugluktuk samples are labeled as CBL and KWWL, respectively. Indicator species are indicated with an asterisk.

The microbial community composition of the Baker Lake WSP samples was dominated by ASVs that affiliated with Acinetobacter and *Pseudomonas*, which together made up ∼75% of the 16S rRNA gene profiles (Fig. 4). In general, few ASVs at >0.2% abundance were common between upstream reference sites, downstream sites, and other WSP sites. For example, for the first time point, samples from the first receiving body of water, Lagoon Lake, did not contain the ASVs associated with these two genera. Although the second time point samples from Lagoon Lake contain one of the ASVs affiliated with Acinetobacter and one affiliated with *Pseudomonas*, these ASVs are found at low abundances. Lagoon Lake also receives water from upstream lakes and is expected to contain microbial inputs from both the WSP and freshwater sources. However, the microorganisms detected in the WSP did not persist at high relative abundance in Lagoon Lake, suggesting attenuation by dilution or competition by microorganisms that are naturally present in the receiving waters.

Finger Lake, Airplane Lake, Baker Lake, and upstream reference lakes also contained very few ASVs at >0.2% abundance in common with the WSP (Fig. 4). Several samples from these lakes had no ASVs in common with the WSP at >0.2% abundance and were therefore not displayed. For the first time point, only 8 ASVs were shared between these lake samples and the WSP samples, which increased to 14 ASVs for the second time point, albeit at <1% abundance. Five of the shared ASVs were detected in upstream reference lake samples at >0.2% abundance, indicating that these microorganisms may be naturally present in the environment. An ASV associated with *Janthinobacterium* was detected at up to ∼35% abundance in an upstream reference lake sample at the first time point but decreased to 2% at the second time point. Several strains of *Janthinobacterium* have been previously identified as psychrotolerant and psychrotrophic (30, 31), which would be consistent with their persistence at high abundance while water temperatures were relatively low.

Despite having similar microbial community profiles to the Baker Lake WSP at the phylum level (Fig. 3), samples from the WSPs in Cambridge Bay and Kugluktuk had very few ASVs at >0.2% abundance in common with the Baker Lake WSP (Fig. 4). The only ASV shared by all three WSPs was related to *Trichococcus*, which was also previously found in influent wastewater in an Arctic region in Finland (28). The Kugluktuk WSP samples shared ASVs associated with the *Acidovorax* and *Rhodoferax* genera. Both of these genera were found at up to ∼15% abundance and were previously detected in samples from municipal WWTPs (32–34). The Cambridge Bay WSP first time point samples shared an ASV associated with *Psychrobacter* with the Baker Lake WSP samples. This ASV was detected at an abundance of ∼83% in the Cambridge Bay WSP. Although *Psychrobacter* species are rare in wastewater environments, they are also generally psychrophilic (35), which may have allowed them to dominate the Cambridge Bay WSP due to the extreme cold. This ASV was not detected in the second time point Cambridge Bay WSP samples, which were collected following WSP discharge. Decreased WSP depth was likely associated with a temperature increase, which may have stimulated the growth of other microorganisms that displaced the *Psychrobacter* representatives.

### Consistent wastewater indicator taxa affiliated with waste stabilization basin samples.

An indicator species analysis (36) identified 46 highly specific wastewater indicator ASVs with an indicator value of >0.9 and a *P* value of <0.05 for the Baker Lake WSP and Lagoon Lake samples. Of these indicator ASVs, 13 were detected at >0.2% abundance in the Baker Lake WSP samples (Fig. 4). This includes the ASVs associated with *Acidovorax*, *Brevundimonas*, *Psychrobacter*, and *Trichococcus*, which were also detected in WSP samples from Cambridge Bay and/or Kugluktuk at >0.2% abundance. The ASV associated with the genus *Trichococcus* was the only indicator ASV that was detected in samples from all three WSPs and Lagoon Lake at >0.2% abundance. This ASV was also detected in a single sample (2ACOB; “second time point Airplane Creek Outlet, replicate B”) at >0.2% abundance. Members of the *Trichococcus* genus are common wastewater pathogens previously identified in Arctic wastewater and tundra soils (28). Thus, it is possible that nutrient-rich conditions of the wastewater allowed members of this genus to persist in the Arctic WSPs, albeit at low abundances.

**FIG 5:**
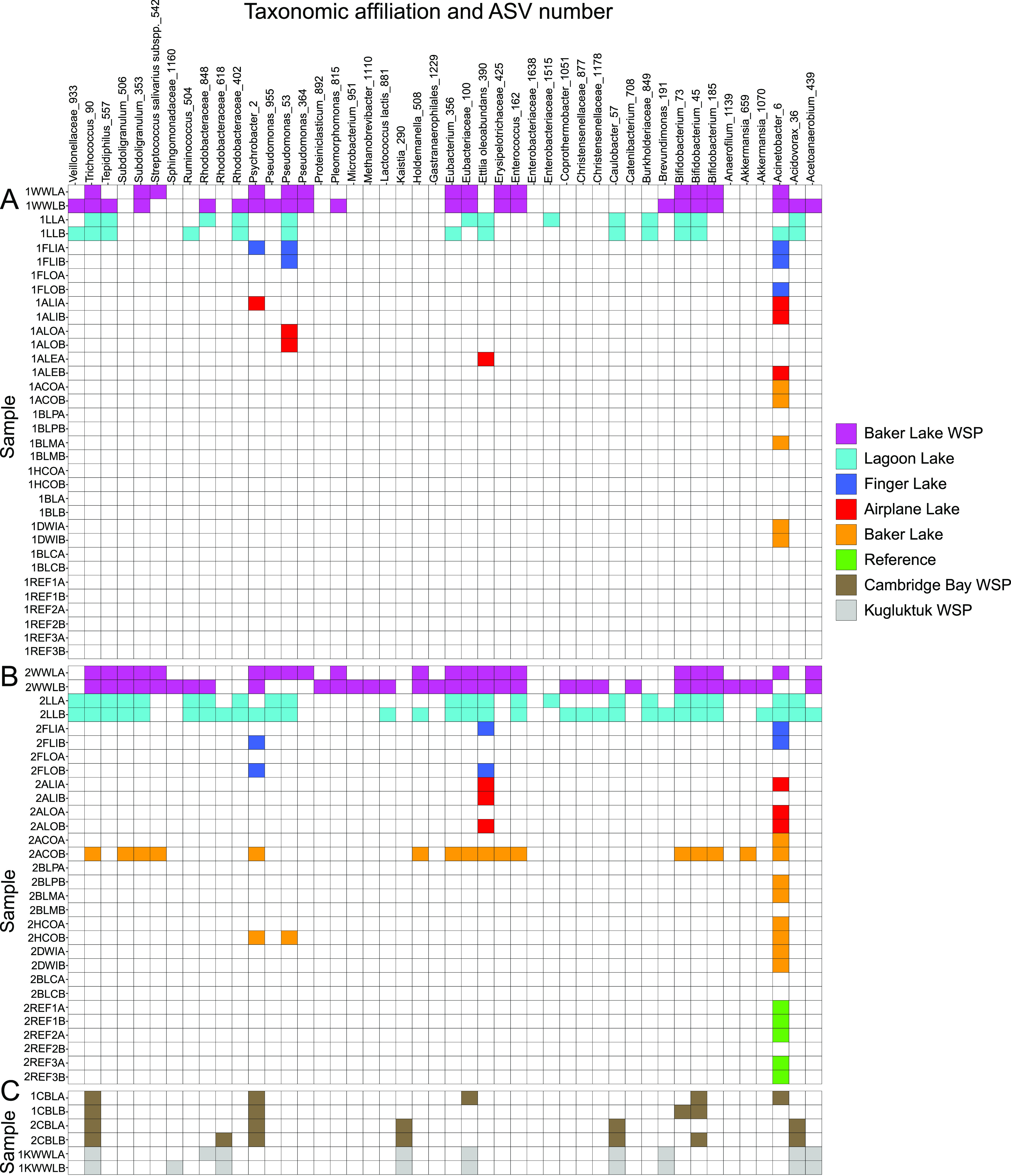
Presence/absence heatmap of wastewater indicator amplicon sequence variants (ASVs) at all sites in Baker Lake, Nunavut, Canada. (A) Baker Lake, 13 to 16 July 2018. (B) Baker Lake, 22 to 24 July 2018. (C) Cambridge Bay and Kugluktuk. Baker Lake sample names are coded by sites on Fig. 1, with prefixes 1 and 2 indicating sampling time and suffixes A and B indicating sample replicate. Cambridge Bay and Kugluktuk samples are labeled as CBL and KWWL, respectively. Boxes are filled in if the ASV had at least 10 reads for the site sample.

Only four of the indicator ASVs were detected in samples from multiple sites downstream of Lagoon Lake with >10 reads per sample (Fig. 5), including ASVs associated with Acinetobacter, Ettlia oleoabundans, *Pseudomonas*, and *Psychrobacter*. All other indicator ASVs were not detected in any downstream sites other than one sample (2ACOB), which was likely contaminated during sample processing because it contained 16 out of 46 indicator ASVs, whereas its replicate (2ACOA) only contained 1 indicator ASV. The ASV associated with Acinetobacter was also detected in reference lake samples during the second time point with >10 reads but only one sample (2REF1B) had >0.2% abundance. Although several species within the Acinetobacter genus are human pathogens, they are also relatively ubiquitous bacteria (37). This ASV cannot be linked directly with wastewater because it was also found at reference sites, which should have no wastewater input. The nutrient-rich environment of the WSP and psychrotolerant adaptations of the species itself may have enhanced the ability of this environmental organism to grow and persist in the Baker Lake WSP at >50% abundance.

### Metagenomic sequence analysis of WSP samples.

A subset of sample sites was used for metagenome sequencing, including each of the three WSPs, Lagoon Lake, the outlet sites of Finger Lake and Airplane Lake, the drinking water intake (DWI) site in Baker Lake, and reference lakes 1 and 2. All sampling sites were sequenced in duplicate at all available time points. A single representative site for each lake was selected, with reference lakes 1 and 2 used because their microbial community profiles were most similar to Baker Lake. Raw reads were analyzed using a hidden Markov model (HMM) approach for the identification of taxa based on the *rpoB* gene. Reads were also assembled into metagenome-assembled genomes (MAGs), and taxonomy was assigned to each MAG. These two data sets, along with the 16S rRNA gene profiles were compared.

Similar proportions of phyla for the same samples occurred in each of the three data sets (Fig. S1). This pattern was less apparent in the MAG taxonomic assignments, presumably because many metagenomic reads were not assigned to bins and were considered “unclassified” (Fig. S1). However, similar proportions of the phyla were still observed when comparing MAG taxonomic assignments to the other two data sets. *Proteobacteria* was the dominant phylum in the Baker Lake WSP across all three data sets. Both *Bacteroidetes* and *Actinobacteria* were highly abundant in the 16S rRNA gene and *rpoB* gene taxonomic assignments but were underrepresented in the MAG taxonomic assignments, likely due to low proportions of mapped reads. Based on *rpoB* gene taxonomic classifications, the *Actinobacteria* phylum was more prominent than suggested by the 16S rRNA gene data set. For example, in the first Baker Lake time point, the *Actinobacteria* had a relative abundance of ∼5% in sample 1ALOA (“first time point Airplane Lake Outlet, replicate A”) based on the 16S rRNA gene (Fig. S1A). However, this relative abundance increased to ∼30% in the same sample based on the *rpoB* genes (Fig. S1B).

A Mantel test using distance matrices for all samples from each data set determined that the *rpoB* data set and 16S rRNA gene data set were most similar, producing statistically significant observed correlations (Table S2). The MAG data set had weaker correlations with the *rpoB* and 16S rRNA gene data sets, but they were still considered significantly positive correlations. Again, this is likely due to the underrepresentation of taxa in the MAG data set as a result of fewer reads mapping to bins. Hierarchical clustering of samples for each data set produced similar clusters among different data sets (Fig. S2). The 16S rRNA gene data set had clustering of samples most similar to the grouping of samples in the distance-based redundancy analysis (db-RDA) (Fig. 2). Replicate samples clustered together consistently. The Baker Lake WSP and Cambridge Bay first time point samples formed a separate cluster from other samples (Fig. S2). Based on hierarchical clustering, Baker Lake and reference lake samples were similar, with no distinct separation between the groups of samples. Lagoon Lake samples clustered with Cambridge Bay second time point samples and Kugluktuk samples, indicating that their microbial compositions were also similar to wastewater.

### Antibiotic resistance genes are enriched in WSP samples.

**FIG 6:**
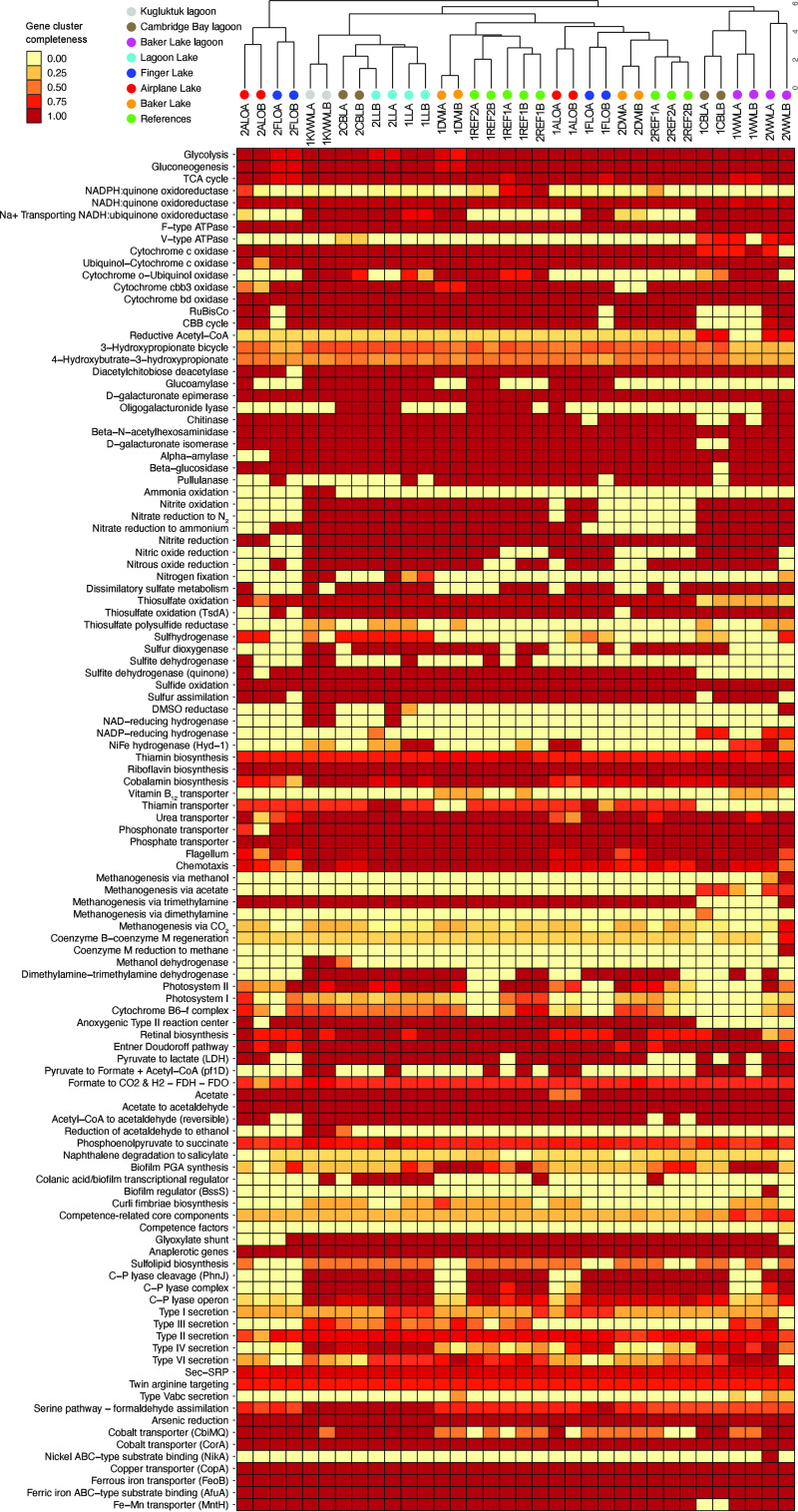
Heatmap of completeness of KEGG pathways based on predicted proteins from metagenome-assembled genomes (MAGs). Baker Lake sample names are coded by sites on Fig. 1, with prefixes 1 and 2 indicating sampling time and suffixes A and B indicating sample replicate. Cambridge Bay and Kugluktuk samples are labeled as CBL and KWWL, respectively.

Metagenome-assembled genomes were further analyzed for completeness of KEGG pathways. The functional profiles of the samples were broadly similar across all samples (Fig. 6). Wastewater samples formed two clusters, with the Baker Lake WWL and two Cambridge Bay WWL samples in one group and the Kugluktuk WWL, Cambridge Bay WWL, and Lagoon Lake samples in a second group. There were few consistent differences in gene cluster completeness between the two WSP clusters and downstream and reference lakes. Overall, the functional composition of all samples appears to be similar despite the differences in taxonomic composition.

Wastewater is often considered to be a hot spot for antibiotic resistance gene dissemination. Studies calculating absolute abundance of ARGs using quantitative PCR (qPCR) have demonstrated that effluent wastewater had higher levels of ARGs than sites upstream of WWTPs (38–41). Another study, by Fitzpatrick and Walsh (42), used metagenomic techniques to demonstrate that environmental water samples contained fewer ARGs than WWTP sludge samples. Although metagenomic techniques are limited by the bias of DNA extraction methods, availability of database sequences for comparison, and sequence coverage required for rare bacterial species, these methods are still able to identify the presence of antibiotic resistance genes and compare relative abundances between different microbiomes.

**FIG 7:**
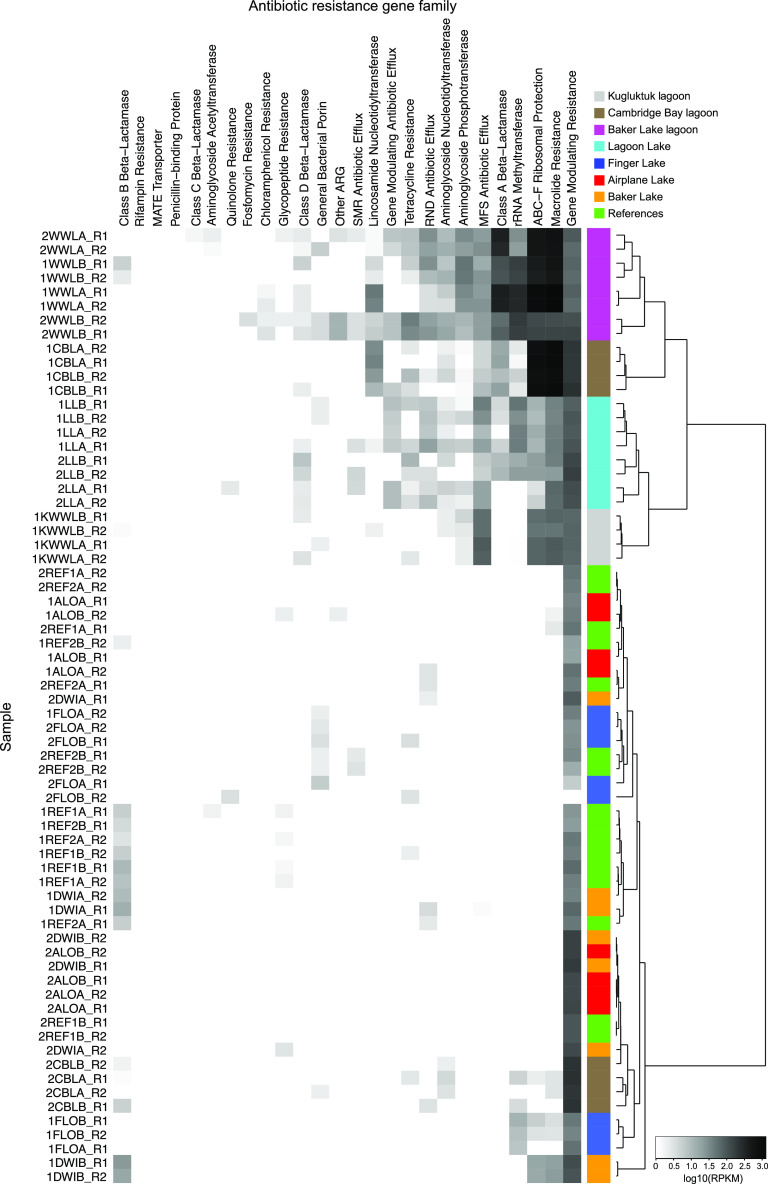
Heatmap of log_10_-transformed RPKM values of antibiotic resistance gene (ARG) families based on raw metagenomic reads. Baker Lake sample names are coded by sites on Fig. 1, with prefixes 1 and 2 indicating sampling time and suffixes A and B indicating sample replicate. Cambridge Bay and Kugluktuk sample names are labeled as CBL and KWWL, respectively. A dendrogram for sample similarity is shown at the top of the figure.

Despite the broad functional similarities observed across metagenomes from all samples (Fig. 6), there were several antibiotic resistance gene families with increased relative abundances in samples from the three WSPs and Lagoon Lake (Fig. 7). The gene families identified were related to macrolide resistance, class A beta-lactamases, ABC-F ribosomal protection, and rRNA methyl transferases. Within these four ARG classes, the gene families with highest abundance all appeared to be related to resistance to macrolide antibiotics, except for a single gene family (CARB-16) that was related to class A beta-lactamases. There were also several antibiotic resistance gene families identified in samples from the WSPs that were also identified in samples from sites downstream of the Baker Lake WSP and upstream reference sites. The genes within these families likely represent the naturally occurring environmental resistance genes.

Macrolide antibiotics are commonly used for the treatment of clinical infections, particularly infections of the upper respiratory tract and skin (43). Although speculative, it is possible that macrolide antibiotic usage by residents may be a source of low concentrations of antibiotics that accumulated in the WSPs over time. These resistance genes were not detected at high abundances in lakes downstream of Lagoon Lake in Baker Lake (Fig. 7), suggesting that the microorganisms carrying these ARGs do not persist in lakes further downstream. The identified ARG families related to macrolide resistance have not been linked with any particular species of bacteria; however, the ARG abundance correlates with the wastewater indicator ASVs identified in this study in that they were not detected in sites downstream of Lagoon Lake.

**Conclusion.** The frigid climate of the Canadian Arctic provides the need for specialized wastewater treatment solutions, often in the form of waste stabilization ponds. The Arctic WSPs studied here contained a microbial community composition dominated by members of the *Proteobacteria*. Despite having this similarity at the phylum level, each WSP had different dominant ASVs, which were also distinct from other Arctic freshwater lake samples. The Baker Lake WSP and downstream samples had considerable temporal and spatial variability, and the microbial community composition appeared to shift considerably during the 10-day period between sampling time points. In addition, samples from sites that were located geographically further from the WSP had less similar microbial community composition to the WSP than samples from sites located closer. Wastewater indicator ASVs were also identified for the Baker Lake WSP, and most of these ASVs were not found in samples from sites downstream of Lagoon Lake, suggesting that wastewater microorganisms are rapidly attenuated with increasing distance from the WSP in this system.

Antibiotic resistant gene families were quantified, and several of these gene families were found to be enriched in samples from the three WSPs. However, on a broad scale, functional profiles of these same appeared to be very similar to all of the other samples. In particular, ARG families related to macrolide resistance were highly abundant in the three WSPs, which may be related to the use of antibiotics within the local community. The abundant ARG families were not detected at high abundances in downstream and reference sites, suggesting that ARB from the WSP do not persist in lakes downstream of Lagoon Lake. Although specific ARBs were not identified in this study, it is possible that microorganisms containing ARGs include the wastewater indicator organisms, as these microorganisms were almost exclusively found in the Baker Lake WSP and Lagoon Lake. Overall, this study has provided a baseline characterization of microbial communities in Arctic WSPs, which will aid in the future development of a safe and effective wastewater treatment system for the Baker Lake community.

## MATERIALS AND METHODS

### Study locations.

This study sampled the microbial communities of WSPs and receiving water in three communities in Nunavut, Canada, Baker Lake (64.3176°N, 96.0220°W), Cambridge Bay (69.1169°N, 105.0597°W), and Kugluktuk (67.8252°N, 115.0966°W).

Baker Lake is an inland community in Nunavut, located west of Hudson’s Bay in the Kivalliq region. The Baker Lake WSP consists of a single WSP located approximately 1 km north of the community. Wastewater is collected and discharged into the WSP year-round, resulting in the accumulation of wastewater into an ice block over the 9-month period when temperatures are subzero. The ice typically thaws in early June, allowing the water to flow into Lagoon Lake. From there, the water flows into Finger Lake, followed by Airplane Lake, before finally discharging into Baker Lake (Fig. 1). Each of these lakes, along with three upstream reference lakes were sampled at two time periods from 13 to 16 July 2018 and 22 to 24 July 2018, with the exception of the ALE and BL sites, which were only sampled within the first time period. The initial spring thaw occurred 2 to 3 weeks before the first set of samples was collected.

Cambridge Bay is located on Victoria Island in the Kitikmeot region of Nunavut. Wastewater is dumped into an engineered WSP, located approximately 1 km northeast of the community, where primary treatment occurs. The wastewater is discharged once a year every summer, when it passes through a natural tundra wetland before discharging into Cambridge Bay. Samples were taken from the WSP on 4 July and 25 July 2018.

Kugluktuk is located at the mouth of the Coppermine River in the Kitikmeot region of Nunavut. Wastewater is dumped into a single-cell, lined sewage lagoon. The wastewater is discharged once a year from the northwest corner of the lagoon. The wastewater then passes through a natural tundra wetland and flows into Coronation Gulf. Samples were taken from the WSP at a single time point on 16 August 2018.

### Sample collection.

Water samples were collected ∼4 to 5 m from the shoreline using a telescoping swing sampler and attached bottle to avoid disturbance of sediments. Samples were collected in duplicate from the top 10 cm of the water column. Water was filtered through a sterile 0.22-μm Sterivex-GV pressure filter unit (EMD Millipore Corporation, MA, USA) using a 60-ml Luer-Lok tip syringe (Becton, Dickinson, NJ, USA). Filtration was done until the filter became plugged or a maximum of 500 ml was filtered. Water was then expelled from the filter unit, and samples were kept in a cooler and surrounded by frozen ice packs until delivered to the lab. Once received by the lab, samples were stored at −20°C.

### DNA extraction.

Filters were removed aseptically from filter housing units and cut in half. One half was used for DNA extractions using the PowerSoil DNA isolation kit (Mo Bio, CA, USA) following the manufacturer’s instructions, and the other half was frozen as a backup. PowerBead tubes were heated at 70°C for 10 min prior to bead beating with a FastPrep-24 device (MP Biomedicals, CA, USA) at 5.5 m/s for 45 s. Extraction kit negative controls were processed alongside samples.

### 16S rRNA gene amplicon library preparation and sequencing.

Using a modification of a previously published amplification and sequencing method (44), the V4-V5 region of the 16S rRNA gene was amplified from genomic DNA extracts using primers 515F-Y/926R (45, 46). Samples were randomly assigned barcodes. Triplicate PCR products were pooled, and DNA for each sample was quantified using a NanoDrop 2000c spectrophotometer using absorbance at 260 nm. The DNA was then relatively quantified using gel electrophoresis. DNA quantification estimates were used to normalize the DNA so that approximately equal amounts of DNA from each sample were combined and sequenced in order to obtain a similar number of reads per sample. Nontemplate negative controls were added as 5 μl volumes, and positive controls, containing equal amounts of Thermus aquaticus and Aliivibrio fischeri were added at half the amount of the samples. The 16S rRNA gene amplicon library was purified and prepared for sequencing on a MiSeq device (Illumina, CA, USA) using the provided HT1 buffer to dilute the library to about 6 pM. Paired-end sequencing (2 × 250 bases) was performed on a MiSeq device, generating ∼18 million paired-end reads.

### Taxonomic profiling and beta diversity analysis.

Paired-end reads were demultiplexed using the MiSeq Reporter software. Subsequent FASTQ files were imported into QIIME 2 (47), and adapter and primer sequences were removed using Cutadapt (48). Reads were then trimmed, denoised, dereplicated, and merged using DADA2 (49) within QIIME 2. Reads were trimmed based on a minimum quality score of 25 for the nucleotide position. This produced a feature table containing amplicon sequence variants (ASVs). The April 2018 SILVA (50) release 132, 97% taxonomy classification for the 16S rRNA gene, was used to train the naive Bayes classifier, and taxonomy was assigned to sequence variants within the feature table. The feature table was collapsed to the phylum level, and the uncollapsed ASV table was rarefied to a sampling depth based on the sample with the lowest number of reads (Data Set S1). A beta diversity analysis was conducted with scripts contained within the QIIME 2 pipeline. Phylogenetic trees were created using the ASV table, and beta diversity analysis was performed using the same sampling depth as for rarefaction. Principal-coordinate analysis (PCoA) was performed using the weighted UniFrac distance metric. A distance-based redundancy analysis (ds-RDA) was performed using the PCoA results using the vegan package in R (51).

### Indicator species analysis.

Rarefied ASV tables exported from QIIME 2 were used for an indicator species analysis, using only Baker Lake WSP samples, Lagoon Lake samples, and reference lake samples. The Baker Lake WSP and Lagoon Lake samples were assigned to one group, and reference lake samples were assigned to another group. Indicator values for each ASV within these samples were calculated using the multipatt function from the indicspecies R package (52); 1,000 permutations were tested to determine statistical significance. The ASVs with an indicator value (IndVal) of at least 0.9 and *P* value of < 0.05 were selected as indicator ASVs.

### Metagenome sequencing.

Genomic DNA was aliquoted for a subset of samples. Each sample location selected was sequenced in duplicate for all time points available. Library preparation, quality control, and whole-metagenome shotgun sequencing were performed by the Farncombe Metagenomics Facility at McMaster University on a HiSeq 1500 device (Illumina), generating a total of ∼500 million paired-end reads.

### HMM taxonomic profiling.

Raw metagenomic reads were analyzed using a development version of MetAnnotate (53) (available at https://github.com/MetAnnotate/MetAnnotate/tree/develop, commit ID: 6e92c0e). A hidden Markov model (HMM) for the gene for the beta subunit of bacterial RNA polymerase (*rpoB*) was downloaded from the TIGRFAMs database (54) and was used to profile the metagenomes by assigning taxonomy to reads identified as hits to the HMM. Forward and reverse reads were analyzed separately using HMM E values of ≤1e–3 and RefSeq HMM E values of ≤1e–6. Default values were used for all other parameters.

### Metagenome assembly and binning.

Metagenomic reads were processed using the ATLAS pipeline (release 2.0.6), which performs quality control, assembly, annotation, binning, and read mapping (55). Quality control was performed with the BBtools suite (56) tools to eliminate adapters, remove PCR duplicates, and then trim and filter reads based on quality scores and lengths of reads. Paired-end reads were assembled using the metaSPAdes (57) assembly tool. Prodigal (58) was used to predict n reading frames (ORFs) in contigs, and translated gene products were mapped to the eggNOG (59) catalogue using DIAMOND (60). Taxonomy was assigned using BAT (61), mapping genes to the GenBank protein database (62). Contigs were then binned using MetaBAT2 (63) and MaxBin2 (64), followed by DASTool (65) as a final binner to dereplicate, aggregate, and score the bins and create high-quality metagenome assembled genomes (MAGs). Because some of the same genomes were identified in multiple samples, resulting in multiple bins for the same MAG, dRep (66) was also used to select the best bin for each MAG.

### Mantel test and hierarchical clustering.

The ASV tables, *rpoB* tables, and MAG tables were compared to one another with a Mantel test to assess correlations between the three data sets. Bray-Curtis dissimilarity matrices were produced for each of the three tables in R using the vegdist function in the vegan package (51). The Mantel test was then performed on each pair of matrices using the mantel.rtest function from the ade4 R package (67), with 1,000 permutations to verify statistical significance. Hierarchical clustering was then performed on each distance matrix using the hclust function in the stats R package (68). The group average agglomeration method was used to calculate the distance between groups.

### Functional profiling of assembled metagenomes and identification of ARGs.

KofamScan (69) was used to assign KEGG ontology to the predicted proteins of MAGs. The output file from KofamScan was then parsed using KEGG-Decoder (70) to identify the completeness of each identified KEGG pathway in each sample. Forward and reverse reads from each set of paired-end metagenomic read files were analyzed separately to identify and quantify antibiotic resistance genes. This was done using the precomputed 2017 Antibiotic Resistance Factors marker collection from the ShortBRED (71) documentation pages (available at http://huttenhower.sph.harvard.edu/shortbred). Gene families were grouped manually into larger groups based on information from the Comprehensive Antibiotic Resistance Database (CARD) (72).

**Data availability.** Metagenome and 16S rRNA gene amplicon sequences are available in the Sequence Read Archive (SRA) at NCBI under BioProject accession number PRJNA600216.

## Supplementary Material

Supplemental file 1

Supplemental file 2
