# Supplementary material for: Monitoring Microbial Populations and Antibiotic Resistance Gene Enrichment Associated with Arctic Waste Stabilization Ponds
Source: Appl Environ Microbiol. 2021 Mar 11;87(7):e02914-20. doi: 10.1128/AEM.02914-20 (PMC8091602; doi:10.1128/AEM.02914-20)
Supplement: Supplemental file 1 [file AEM.02914-20-s0001.pdf]

Figure S1. Relative abundance of phyla present at metagenome sequenced sites from Baker Lake, Cambridge Bay, and Kugluktuk. (A) Baker Lake, July 13-16, 2018. (B) Baker Lake, July 22-24, 2018. (C) Cambridge Bay and Kugluktuk samples. Sample names correspond to sites indicated in Figure 1. Letters A and B at the end of sample names refer to replicates at the same site and time point. R1 and R2 refer to forward and reverse reads, respectively. Site locations are indicated above bars.

Figure S2. Dendrograms based on hierarchical clustering done using the average agglomeration method and Bray-Curtis distances for (A) taxonomic assignment to 16S rRNA gene amplicon sequences (B) taxonomic assignment to sequences corresponding to the *rpoB* hidden Markov model (HMM) and (C) taxonomic assignment to metagenome-assembled genomes (MAGs). Baker Lake samples names are coded by sites in Figure 1, with prefix 1 and 2 indicating sampling time and suffix A or B indicating sample replicate. Cambridge Bay and Kugluktuk samples are labelled as CBL and KWWL, respectively.

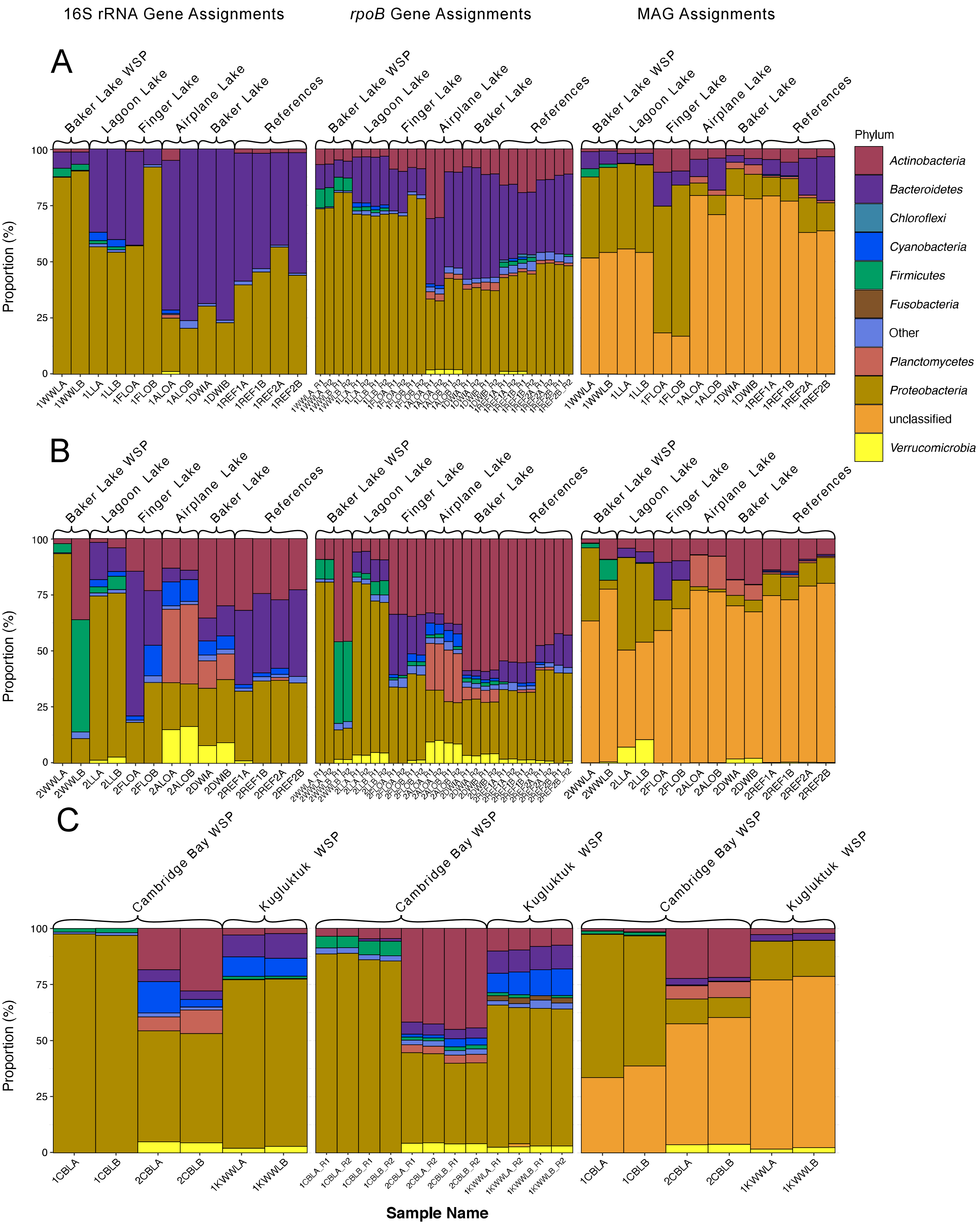
16S rRNA Gene Assignments
rpoB Gene Assignments
MAG Assignments
A
B
C
Baker Lake WSP
Lagoon Lake
Finger Lake
Airplane Lake
Baker Lake
References
Cambridge Bay WSP
Kugluktuk WSP
Proportion (%)
100
75
50
25
0
1WWLA
1WWLB
1LLA
1LLB
1FLOA
1FLOB
1ALOA
1ALOB
1DWIA
1DWIB
1REF1A
1REF1B
1REF2A
1REF2B
2WWLA
2WWLB
2LLA
2LLB
2FLOA
2FLOB
2ALOA
2ALOB
2DWIA
2DWIB
2REF1A
2REF1B
2REF2A
2REF2B
1CBLA
1CBLB
2CBLA
2CBLB
1KWWLA
1KWWLB
1CBLA_R1
1CBLA_R2
1CBLB_R1
1CBLB_R2
2CBLA_R1
2CBLA_R2
2CBLB_R1
2CBLB_R2
1KWWLA_R1
1KWWLA_R2
1KWWLB_R1
1KWWLB_R2
Sample Name
Phylum
Actinobacteria
Bacteroidetes
Chloroflexi
Cyanobacteria
Firmicutes
Fusobacteria
Other
Planctomycetes
Proteobacteria
unclassified
Verrucomicrobia

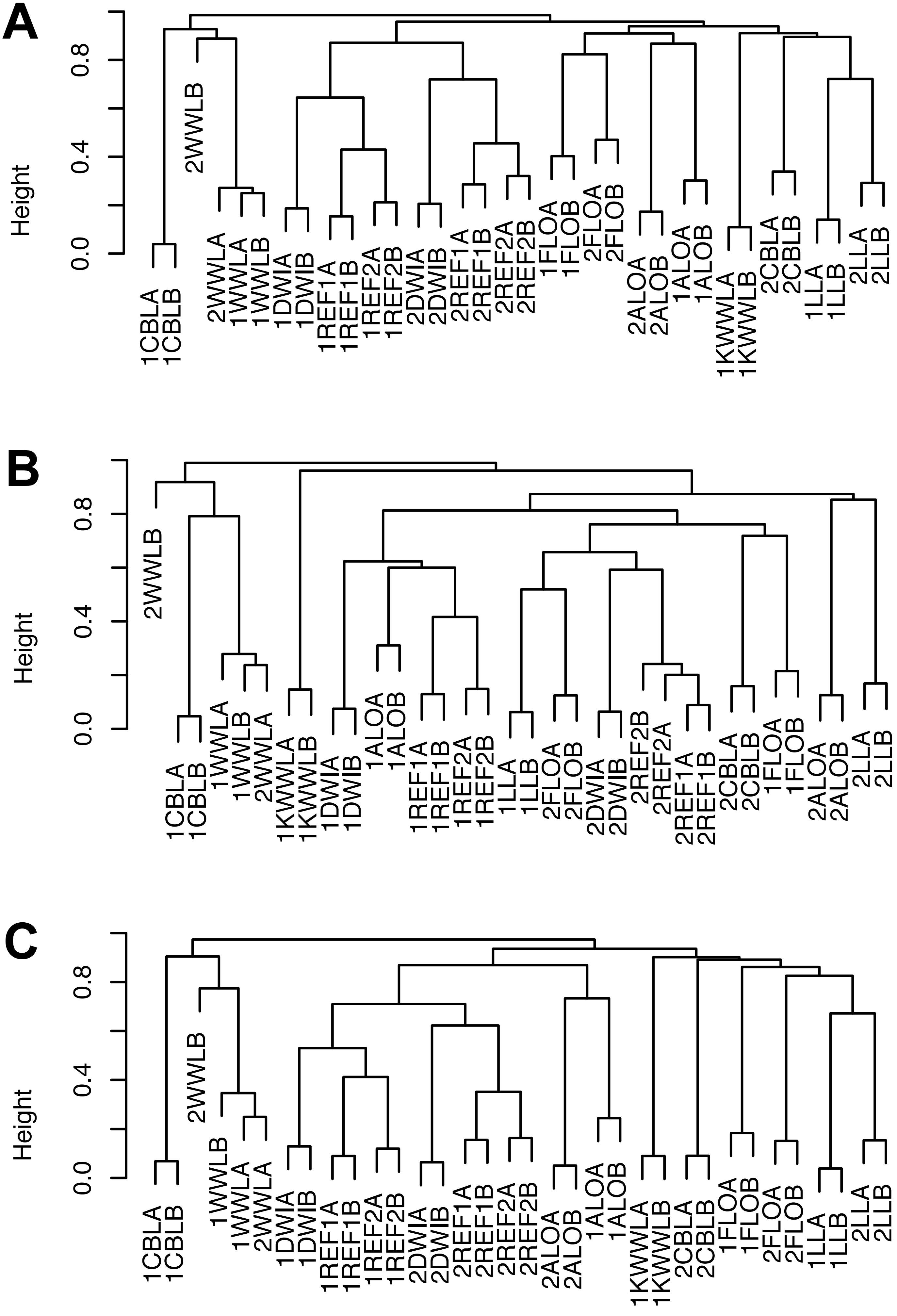
A
Height
0.0
0.4
0.8
1CBLA
1CBLB
2WWLB
2WWLA
1WWLA
1WWLB
1DWIA
1DWIB
1REF1A
1REF1B
1REF2A
1REF2B
2DWIA
2DWIB
2REF1A
2REF1B
2REF2A
2REF2B
1FLOA
1FLOB
2FLOA
2FLOB
2ALOA
2ALOB
1ALOA
1ALOB
1KWWLA
1KWWLB
2CBLA
2CBLB
1LLA
1LLB
2LLA
2LLB
B
Height
0.0
0.4
0.8
2WWLB
1CBLA
1CBLB
1WWLA
1WWLB
2WWLA
1KWWLA
1KWWLB
1DWIA
1DWIB
1ALOA
1ALOB
1REF1A
1REF1B
1REF2A
1REF2B
1LLA
1LLB
2FLOA
2FLOB
2DWIA
2DWIB
2REF2B
2REF2A
2REF1A
2REF1B
2CBLA
2CBLB
1FLOA
1FLOB
2ALOA
2ALOB
2LLA
2LLB
C
Height
0.0
0.4
0.8
1CBLA
1CBLB
2WWLB
1WWLB
1WWLA
2WWLA
1DWIA
1DWIB
1REF1A
1REF1B
1REF2A
1REF2B
2DWIA
2DWIB
2REF1A
2REF1B
2REF2A
2REF2B
2ALOA
2ALOB
1ALOA
1ALOB
1KWWLA
1KWWLB
2CBLA
2CBLB
1FLOA
1FLOB
2FLOA
2FLOB
1LLA
1LLB
2LLA
2LLB

Table S1. Environmental data from sites in Baker Lake, Nunavut. Site names correspond to sample sites in Figure 1.

Site Name	Latitude	Longitude	Temperature (°C)		pH		Conductivity (µS/cm)		TDS (mg/L)		Salinity (ppt)		DO (mg/L)	
			July 13-16	July 22-24	July 13-16	July 22-24	July 13-16	July 22-24	July 13-16	July 22-24	July 13-16	July 22-24	July 13-16	July 22-24
**REF1**	64.33715	-95.96131	12.6	15.2	7.07	7.59	81.6	84.1	59.0	59.7	0.04	0.04	9.5	8.2
**REF2**	64.336836	-96.016599	14.8	16.1	7.38	7.72	112.3	60.4	80.0	43.0	0.06	0.03	9.8	9.1
**REF3**	64.33707	-96.02652	16.1	16.7	7.62	8.06	101.0	73.8	71.4	52.3	0.05	0.04	9.3	9.7
**DWI**	64.31557	-96.01727	10.1	11.1	6.94	7.31	108.2	31.0	77.1	22.0	0.05	0.02	10.9	11.0
**HCO**	64.31519	-96.01374	6.1	11.1	7.14	7.59	231	48.6	165	34.7	0.12	0.02	11.8	11.4
**BLM**	64.311844	-95.993918	6.1	9.5	6.96	7.47	158.6	31.0	113	22.0	0.08	0.02	12.4	10.9
**BLP**	64.30668	-95.96240	7.0	9.9	7.02	7.51	142.1	33.0	101	23.7	0.07	0.02	12.8	10.3
**ACO**	64.30960	-95.97852	8.3	9.2	7.04	7.48	149.7	34.3	107	24.4	0.08	0.02	12.3	11.6
**ALO**	64.31702	-95.97436	14.6	12.9	9.06	7.62	86.4	79.7	61.2	56.5	0.04	0.04	10.5	11.3
**ALI**	64.32044	-95.97424	16.2	14.5	9.06	7.73	94.6	82.7	67.4	58.8	0.05	0.04	10.7	10.5
**FLI**	64.32967	-95.99605	17.2	15.0	9.04	7.16	166.5	202	119	143	0.08	0.10	9.6	7.7
**FLO**	64.32796	-95.98515	17.4	15.1	9.64	9.13	164	215	117	152	0.08	0.11	10.1	10.8
**LL**	64.33113	-96.00263	16.1	17.6	9.40	8.95	188.0	289	134	205	0.09	0.14	14.3	10.3
**WWL**	64.32971	-96.00595	18.7	21.3	7.84	7.79	928	1125	670	794	0.47	0.56	3.3	6.7
**BLC**	64.31625	-96.04948	10.3	11.5	7.40	7.52	231	44.5	164	31.6	0.12	0.02	10.9	10.9
**BL**	64.31300	-96.02019	6.2	N/A	7.42	N/A	64.2	N/A	45.5	N/A	0.03	N/A	10.7	N/A
**ALE**	64.32290	-95.95353	14.7	N/A	8.15	N/A	78.6	N/A	55.8	N/A	0.04	N/A	9.7	N/A

Table S2: Mantel test results comparing distance matrices for each pair based on 16S rRNA gene amplicons, taxonomic assignment to sequences corresponding to the *rpoB* hidden Markov model (HMM), and taxonomic assignment to metagenome-assembled genomes (MAGs).

	**Observed Correlation**	***p* value**
16S rRNA gene vs. *rpoB gene*	0.83	0.001
16S rRNA gene vs. MAGs	0.63	0.001
rpoB gene vs. MAGs	0.52	0.001
